# Micro ribonucleic acid-448 regulates zinc finger e-box binding homeobox 1 to inhibit the growth of breast cancer cells and increase their sensitivity to chemotherapy

**DOI:** 10.1016/j.clinsp.2022.100089

**Published:** 2022-07-26

**Authors:** Yizhou Zhou, Li Sun, Yangmei Zhang, Kai Chen

**Affiliations:** a Department of Oncology, the First Affiliated Hospital of Soochow University, Soochow, Jiangsu, China; b Department of Oncology, Xuzhou Central Hospital, Xuzhou, Jiangsu, China

**Keywords:** miR-448, ZEB1, Breast cancer cells, Paclitaxel, 5-Fluorouracil, Chemotherapy Sensitivity

## Abstract

**Objective:**

This study aimed to investigate the effect of Zinc Finger E-box Binding Homeobox 1 (ZEB1) regulation by Micro Ribonucleic acid (miR)-448 on Breast Cancer (BC) cells and their sensitivity to chemotherapy.

**Methods:**

miR-448 and ZEB1 mRNA levels in BC and normal tissues were detected by qPCR, and ZEB1 protein was detected by Western Blotting (WB). The correlation between miR-448 and tumor metastasis, clinical staging, and ZEB1 expression was analyzed. MCF-7 cells were transfected or co-transfected with the miR-448 mimic, oe-ZEB1, or their negative controls. Changes in miR-448 and ZEB1 expression were detected by qPCR and WB. Cell proliferation was determined by CCK-8 assays, invasion changes were analyzed by Transwell assays, and apoptosis was detected by flow cytometry.

**Results:**

miR-448 expression in BC tissues was lower than that in normal tissues, while ZEB1 expression was increased in the former. ZEB1 expression was lower in BC patients with lymph node metastasis than in those without. In patients with clinical stage I–III BC, miR-448 expression decreased with an increase in tumor stage, which was negatively correlated with ZEB1 expression. Upregulation of miR-448 expression can suppress MCF-7 cell proliferation and invasion and promote apoptosis. Upregulation of ZEB1 expression in cells overexpressing miR-448 can partially reverse the inhibition of BC cell growth induced by miR-448. miR-448 can enhance the sensitivity of cells toward paclitaxel and 5-fluorouracil.

**Conclusions:**

miR-448 suppresses cell proliferation and invasion and promotes apoptosis by targeting ZEB1. Moreover, it can increase the sensitivity of cells toward paclitaxel and 5-fluorouracil.

## Highlights

• MicroRNA-448 expression decreased in Breast Cancer (BC) tissues and BC cells.
• Zinc finger E-box Binding Homeobox 1 (ZEB1) was upregulated in BC tissues and cells.
• Upregulation of miR-448 expression could inhibit the malignant behaviors of BC cells.
• Upregulated miR-448 expression could enhance BC cells’ sensitivity to PTX or 5-FU.

## Introduction

Breast Cancer (BC) is the most common malignancy in women.^1^ In China, BC-associated morbidity is high, while BC-related mortality ranks third among tumor-related diseases.^2^ The disease develops and progresses through polygenic, multifactorial, and multi-step complex processes; these processes interact and are mutually regulated.^3^ Although the incidence of BC continues to increase, the mortality rate associated with it is declining.^4^ This may be largely due to the systematic development of treatments, including chemotherapy,^5^,^6^ hormone therapy,^7^ radiotherapy,^8^ and targeted therapy.^9^ Because of the high cost of targeted therapy, chemotherapy is still the first choice of treatment in many developing and underdeveloped countries. However, drug resistance is the main cause of chemotherapy failure.^10^ Other factors, such as metastasis, are also responsible for chemotherapy failure; in addition, the long-term survival rate of BC patients is not ideal.^11^ The discovery period is relevant to the prognosis.^12^ Clinical studies have confirmed that the 5-year survival rate of early-stage BC patients after treatment is as high as 70%–90%, while for patients with advanced-stage BC, the rate can be as low as < 15% due to the proliferation or metastasis of cancer cells.^13^ Therefore, there is an urgent need to develop a new strategy to treat BC to lower the dosage and side effects of chemotherapy drugs and improve their efficacy.

Many studies have confirmed that miRNA expression is relevant to disease development and progression. Many studies have found that there are marked differences in miRNA expression between normal and cancerous cells, and most of these differences are related to the gene loci of cancer cells.^14^, ^15^, ^16^ In addition, miRNA is involved in the process of tumor formation and related drug resistance, which affects the growth of tumor cells and the treatment.^17^ As potential targets, various miRNA have been found to be involved in the sensitivity of BC chemotherapy. For example, it has been found that the miR-326 expression in BC MCF-7 cells resistant to etoposide is down-regulated, and increasing the expression by cell transfection can increase the sensitivity of drug-resistant cells to adriamycin and etoposide.^17^ Another study found that over-expressing miR-451 in BC cell lines can reduce the drug resistance of adriamycin, which leads to the enhancement of cell proliferation and invasion.^18^ In the present study, by overexpressing miR-448, changes in the sensitivity of MCF-7 BC cells to chemotherapeutic drugs were observed, and a novel target for chemosensitivity enhancement was discovered.

## Materials and methods

### Clinical tissues and cell lines

From January 2014 to January 2015, cancer tissues and adjacent normal tissues were collected from 38 primary BC patients admitted to the Breast Surgery Department of our hospital. Among them, one patient had invasive lobular carcinoma, while 37 had invasive ductal carcinoma. There were 25 patients with lymph node metastasis and 13 without. BC staging is based on the standards established by the American Joint Committee on Cancer. In this study, 9 patients were at stage I, 15 at stage II, 11 at stage III, and 3 at stage IV of BC. The collection of all tissue samples was approved by the ethics committee of the First Affiliated Hospital of Soochow University. All study participants provided written informed consent before participating in the study. None of the patients had a history of radiotherapy or chemotherapy before surgery. After fresh tissues were excised during surgery, they were stored in liquid nitrogen for transfer to the laboratory. The patients were followed up for 5 years. Human BC cell lines MDA-MB-231, MCF-7, T47D, DK-BR-3, and breast epithelial cell MCF-10A were provided by the Shanghai Institute of Cell Research, Chinese Academy of Sciences. Those were cultivated in RPMI1640 with 10% FBS at 37°C, 5% CO_2,_ and a saturated humidity incubator.

### qPCR

Total RNA was separated by TRIzol reagent (Takara), and cDNA was synthesized with miScript II RT Kit. The miScript SYBR Green PCR Kit (Qiagen) was applied in real-time quantitative PCR. PCR primers were designed and synthesized by Make Research Easy Co., Ltd. The following primers were used: miR-448, F: 5′-TTATTGCGATGTGTTCCTTATG-3′ and R: 5′-ATGCATGCCACGGGCATATACACT-3′; U6, F: 5′-CTCGCTTCGGCAGCACA-3′ and R: 5′-AACGCTTCACGAATTTGCGT-3′; ZEB1, F: 5′-GCCAATAAGCAAACGATTCTG-3′ and R: 5′-TTTGGCTGGATCACTTTCAAG-3′; GAPDH: F: 5′-CAAGGTCATCCATGACAACTTTG-3′ and R: 5′-GTCCACCACCCTGTTGCTGTAG-3′. U6 was used as an internal reference for miR-448, GAPDH was used as an internal reference for ZEB1, and the 2^−△△Ct^ method was employed for quantitative analysis. All reactions were performed with three negative controls with multiple holes and no template.

### Cell transfection

The logarithmic growth phase cells were inoculated into a 6-hole plate, and miR-448 mimic and its negative control, miR-448 mimic+oe-ZEB1, mimic NC+oe-NC, and miR-448 mimic+oe-NC were transfected into MDA-MB-231 and MCF-7 cells via Lipofectamine 2000 kit.

### WB

Total proteins were extracted from the tissues and cells. The concentration of each sample was measured and adjusted with deionized water to ensure the same loading amount. Next, 10% SDS separation and concentrated gels were collected. The samples were blended with sample buffer, boiled at 100°C for 5 min, cooled in an ice bath, centrifuged, and then added to each lane with a micro-sampler for electrophoresis separation. The gel protein was transferred to the nitrocellulose membrane. Afterward, the cellulose nitrate membrane was closed by 5% skimmed milk powder at 4°C all night. The primary antibody was added: ZEB1 and GAPDH (1:1000, Proteintech) were incubated overnight and washed with PBS (Phosphate Buffer) 3 times at indoor temperature, each time for 5 min. The second antibody of HRP labeled IgG (1:1000, Boster Biological Technology Co. Ltd) was incubated 1h at 37°C. At indoor temperature, PBS buffer solution was cleaned 3 times, 5 min each time. And the membrane was immersed for 1 min in an ECL reaction solution (Pierce, USA). The liquid was removed, closed by cling film, and exposed with X-Ray film in a dark environment. The results were observed after developing and fixing. GAPDH was considered as an internal reference, and the protein imprinted images were analyzed by ImageJ2x software.

### CCK-8 assay

The transfected cells were collected and prepared into a 2 × 10^4^ cells/mL single-cell suspension. The suspension (100 μL/well) was inoculated into the 96-hole plate. Then, the plate was cultivated at 37°C, with 5 multiple wells in each group. Subsequently, 10 μL of CCK-8 solution was added 24h, 48h, 72h, and 96h later, and special attention was paid to avoid bubble formation during sample addition. The culture plates were incubated for 2h at 37°C. Cell absorbance was measured at 450 nm using a microplate reader. The test was conducted three times, and the data were recorded.

Drug experiment: The cells were collected and inoculated into a 96-well plate. Each group had three wells, and a blank control group was also used. Paclitaxel (PTX) or 5-Fluorouracil (5-FU) at final concentrations of 2.5, 5.0, 7.5, 10.0, and 12.5 ng/mL or 10, 20, 30, 40, and 50 μg/mL, respectively, were added after cell attachment. Seventy-two hours after cell treatment, the culture medium was discarded, and 10% CCK-8 solution was supplemented to cultivate for 2h. The absorbance value of magnetic nails at 450 nm of the microplate reader was measured. The IC50 growth inhibition rate of each group = [(average absorbance value of control hole-that of an experimental hole)/that of control hole] × 100%.

### Plate cloning

The cells of each group were inoculated into a 6-well plate in a complete medium containing 10% FBS. Next, they were incubated at 37°C and 5% CO_2_ in a saturated humidity incubator for 2 weeks. When visible clones appeared on the culture dish, the supernatant was discarded, cleaned twice with PBS, and immobilized for 15 min with 4% paraformaldehyde. After the cells were rinsed carefully with PBS twice, they were dyed with an appropriate amount of crystal violet staining solution (Wuhan Google Biological Co., Ltd.) for 10 min, following which the solution was washed off slowly with running water. After the plate was dried, the clone number was counted directly with the naked eye.

### Transwell assay

The upper surface of the Transwell chamber basement membrane was coated with Matrigel matrix glue diluted with serum-free cell culture solution, and each group of cell suspension, which was diluted with 100 μL of serum-free culture medium (approximately 2 × 10^5^ cells), was added. The complete culture solution with 10% serum was supplemented to the lower orifice plate of the chamber. The culture chamber was taken out after 24-hour culture at 37°C. The matrix glue and cells were cleaned using wet cotton swabs and immobilized with 4% paraformaldehyde.

### Apoptosis

The cells from each group were collected. Apoptosis was analyzed using a fluorescein isothiocyanate/Propidium Iodide (PI) kit (BD Biosciences, San Jose, CA, USA) and flow cytometry.

### Dual-luciferase reporter gene assay

The miR-448 and ZEB1 binding sites were predicted using the miRNA database and identified by a dual-luciferase reporter gene assay. The ZEB1 3′-UTR was synthesized and cloned into a plasmid in the psiCHECK reporter (Promega) and verified by DNA sequencing and named Wild-Type ZEB1 (WT-ZEB1). Then, a 3′-UTR Mutant (MUT-ZEB1) was constructed and synthesized through site-directed mutagenesis. MDA-MB-231 and MCF-7 cells were co-transfected with recombinant reporter gene WT, MUT plasmid, and mimic NC, or miR-448 mimic in a 24-well plate. The activity was tested after 48h using a dual-luciferase reporter assay (Promega).

### Statistical methods

The results were assessed using SPSS 22.0, and the measurement data are represented as the mean ± SD. The differences between the two groups were evaluated using the independent samples *t*-test, while differences between multiple groups were assessed using one-way ANOVA. Both groups were examined using Tukey's multiple comparison test. The prognosis of patients with cholangiocarcinoma was examined using Kaplan-Meier analysis. The difference was considered statistically significant at p < 0.05.

## Results

### miR-448 and ZEB1 levels in BC

**Fig. 1:**
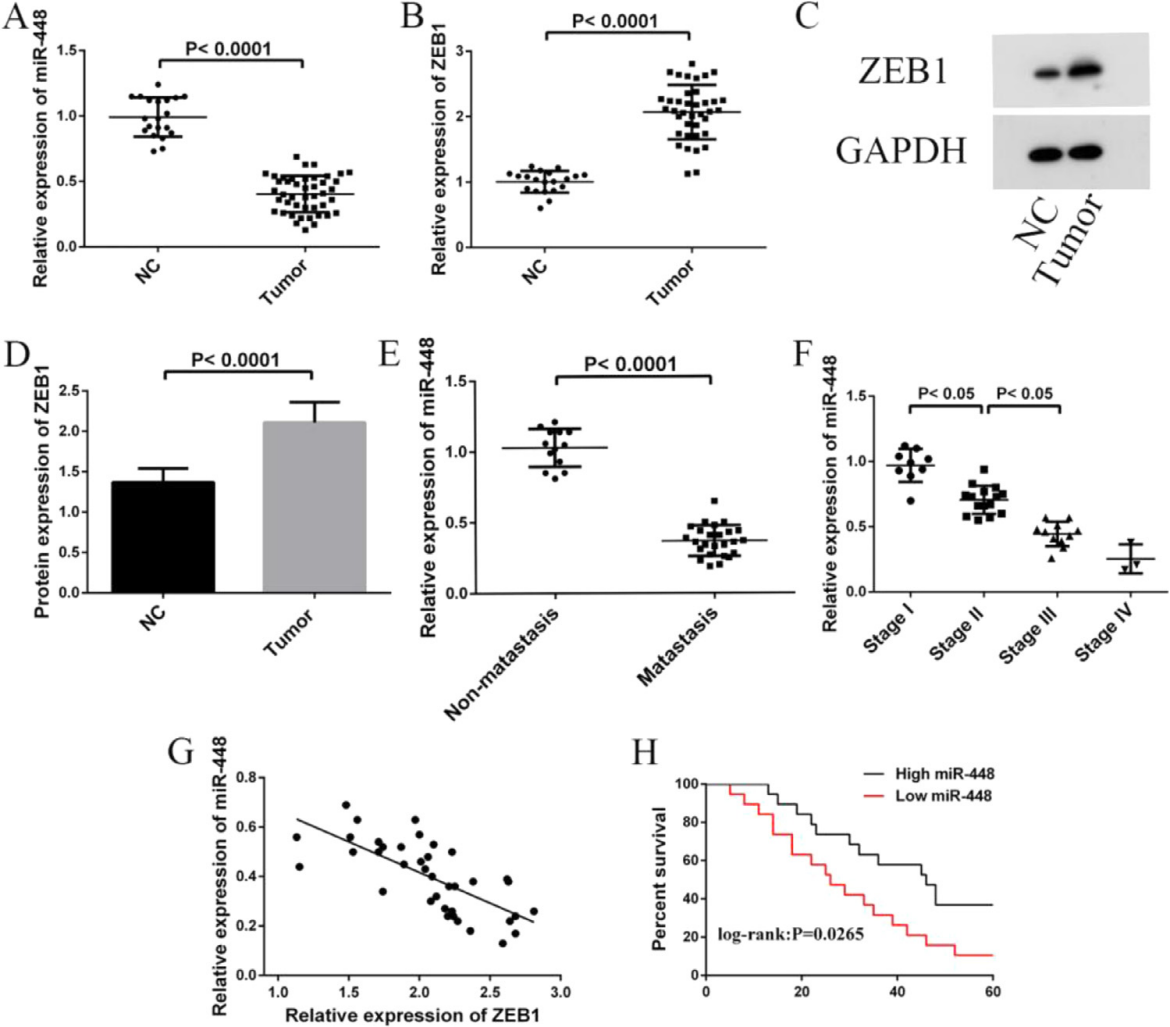
**miR-448 and ZEB1 expression in BC.** (A) Expression of miR-448 in BC; (B) Expression of ZEB1 mRNA in BC; (C) ZEB1 protein expression; (D) Expression of ZEB1 protein in BC; (E) Expression of miR-448 in patients with or without lymph node metastasis; (F) miR-448 expression in BC patients at different stages; (G) Correlation between miR-448 and ZEB1 expression; Correlation was determined using Spearman analysis. and (H) 5-year survival rate of patients with high and low expression of miR-448.

qPCR and WB analysis revealed that miR-448 expression decreased and ZEB1 levels increased in BC tissues (p < 0.0001, Fig. 1A‒1D). Moreover, miR-448 expression was lower in BC patients with lymph node metastasis than in those without (p < 0.0001, Fig. 1E). For patients at different stages, the expression of miR-448 decreased as the stage increased (p < 0.05, Fig. 1F), but stages III and IV showed no obvious differences (p < 0.05). The miR-448 level was negatively correlated with that of ZEB1, suggesting the potential functional relevance between ZEB1 and miR-448 (Fig. 1G). In view of the median level of miR-448, patients were randomized into high and low expression groups. miR-448 level's influence on BC patients’ survival and prognosis was assessed via Kaplan-Meier. It manifested that the 5-year survival rate of those in the low-expression group was lower (p < 0.05, Fig. 1H).

### miR-448 and ZEB1 levels in cells

**Fig. 2:**
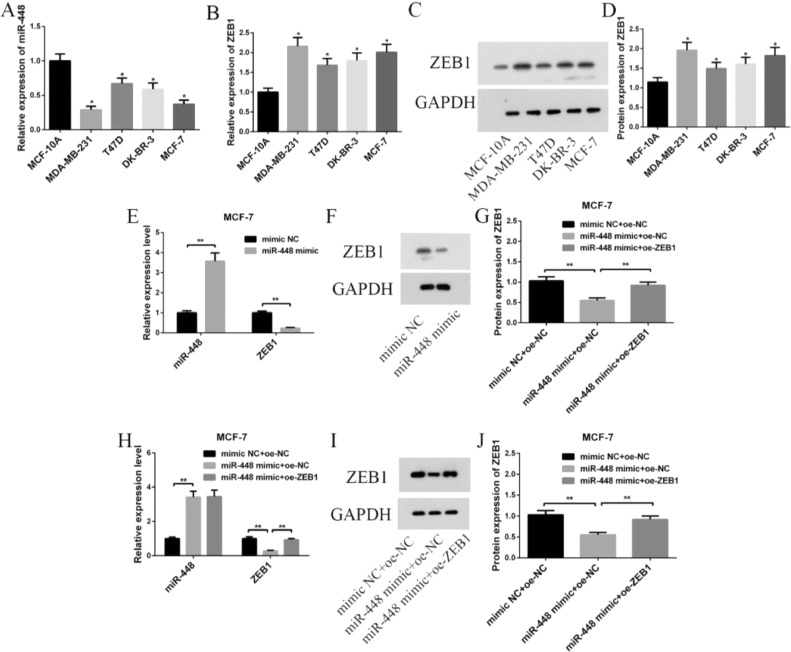
**miR-448 and ZEB1 expression in each cell line.** (A) Expression of miR-448 in each cell line; (B) Expression of ZEB1 mRNA in each cell line; (C) ZEB1 protein band map in each cell line; (D) Expression of ZEB1 protein in various cell lines; (E) Expression of miR-448 and ZEB1 mRNA in MCF-7 cells after transfection; (F) ZEB1 protein banding pattern in transfected MCF-7 cells; (G) Expression of ZEB1 protein in MCF-7 cells after transfection; (H) Expression of miR-448 and ZEB1 mRNA in MCF-7 cells after co-transfection; (I) ZEB1 protein banding pattern in MCF-7 cells after co-transfection; (J) Expression of ZEB1 protein in MCF-7 cells after co-transfection; * Compared with MCF-10A cells, p < 0.05; **p < 0.01.

Compared with human breast epithelial cell MCF-10A, the miR-448 expression in all human BC cells decreased, while the ZEB1 mRNA and protein level increased (p < 0.05, Fig. 2A‒2D). MCF-7 cells were selected as subjects. Compared with mimic NC and mimic NC+oe-NC groups, the miR-448 expression in miR-448 mimic and miR-448 mimic+oe-NC groups increased, while that of ZEB1 mRNA and protein decreased (p < 0.01, Fig. 2E‒12G). Compared with miR-448 mimic+oe-NC group, the miR-448 level in miR-448 mimic+oe-ZEB1 group had no obvious difference, but that of ZEB1 mRNA and protein increased (p < 0.01, Fig. 2H‒2J).

### Effect of miR-448 expression on the biological behavior of BC cells

**Fig. 3:**
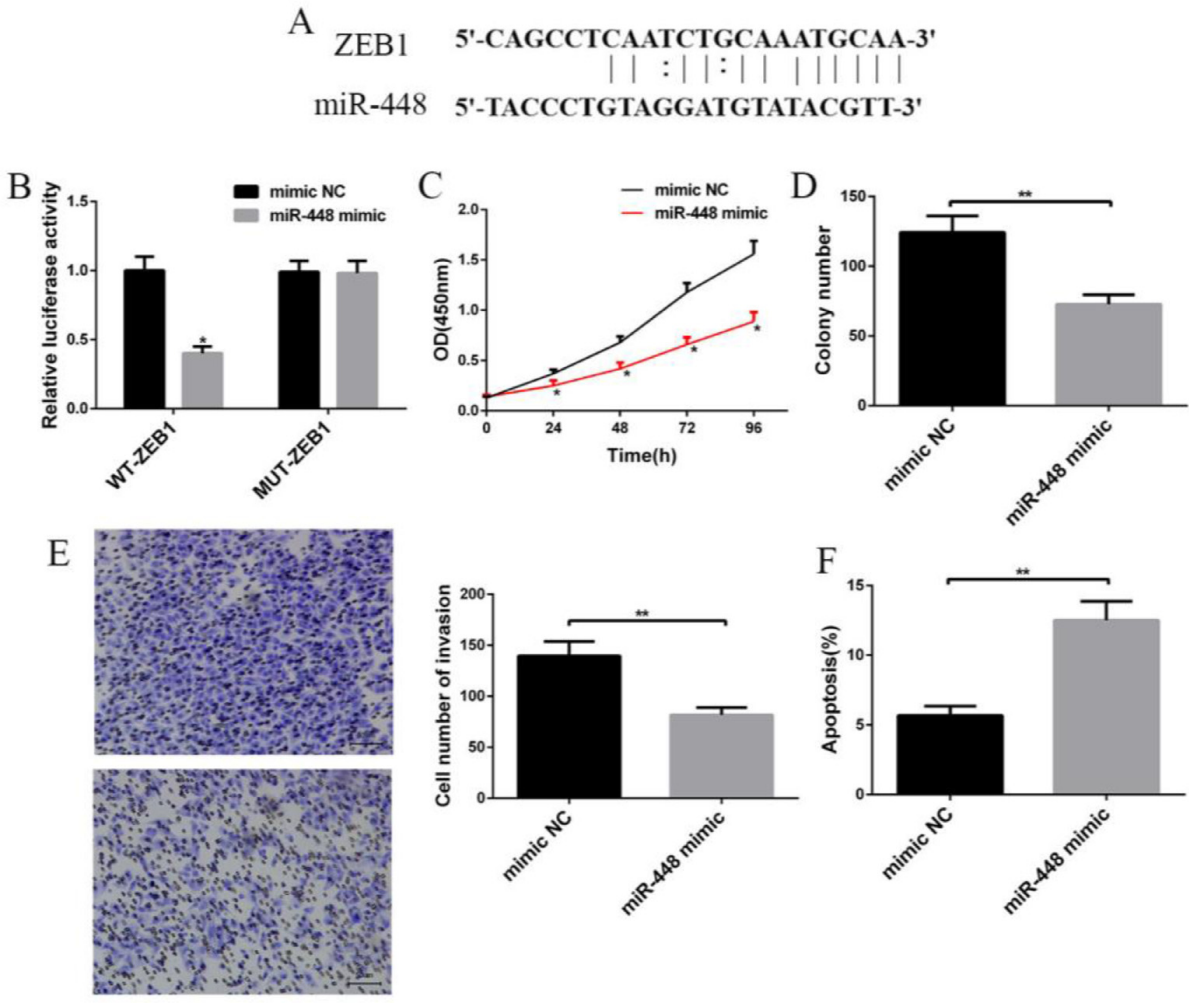
**Effects of miR-448 expression on the biological behavior of BC cells.** (A) Predicted possible binding sites of miR-448 and ZEB1; (B) Dual-luciferase reporter gene assay of MCF-7 cells; (C) Effects of miR-448 on BC cell proliferation; (D) Effects of miR-448 on the plate clone number of BC cells; (E) Effects of miR-448 on the invasion of BC cells; (F) Effects of miR-448 on BC cell apoptosis: * Compared with the control group, p < 0.05; **p < 0.01.

The possible binding sites of miR-448 and ZEB1 were predicted using an online biological prediction tool and were verified using a dual-luciferase reporter gene assay. The results showed that when WT-ZEB1 and miR-448 were co-transfected into MCF-7 cells, the fluorescein activity decreased (p < 0.05, Fig. 3A‒3B), while this activity followed others, co-transfections did not change. Compared with the mimic NC group, cell proliferation activity (p < 0.01, Fig. 3C) and a number of clonal (p < 0.01, Fig. 3D) and invasive cells in the mimic group transfected with miR-448 was reduced (p < 0.01, Fig. 3E), and the apoptosis rate increased (p < 0.01, Fig. 3F) (Fig. 3).

### Effects of miR-448 and ZEB1 expression on the biological behavior of BC cells

**Fig. 4:**
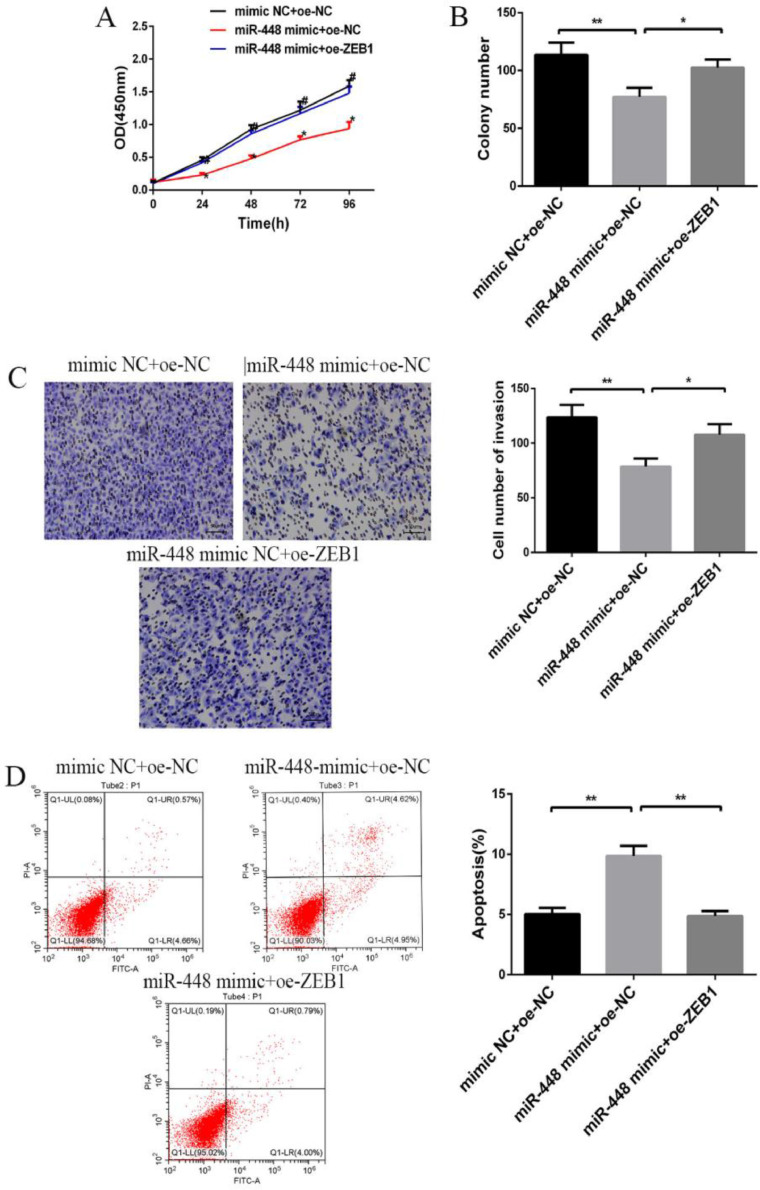
**Effects of miR-448 and ZEB1 expression on the biological behavior of BC cells.** (A) Effects of miR-448 and ZEB1 on BC cell proliferation; (B) Effects of miR-448 and ZEB1 on plate clone number of BC cells; (C) Effects of miR-448 and ZEB1 on BC cell invasion; (D) Effects of miR-448 and ZEB1 on the apoptosis of BC cells; * Compared with the control group, # compared with the miR-448 mimic+oe-NC group, p < 0.05; ** p < 0.01.

Compared with the mimic NC+oe-NC group, the cell proliferation activity, the number of plate clonal and invasive cells in the miR-448 mimic+oe-NC group reduced (p < 0.05, Fig. 4A), and the apoptosis rate rose (p < 0.01, Fig. 4B). Conversely, compared with miR-448 mimic+oe-NC group, the cell proliferation activity of co-transfected miR-448 mimic+oe-ZEB1 group increased (p < 0.05, Fig. 4C), and the number of clonal and invasive cells increased, while the apoptosis rate decreased (p < 0.01) (Fig. 4, Fig. 4D).

### Influence of miR-448 on the sensitivity of BC cells to PTX and 5-FU

**Fig. 5:**
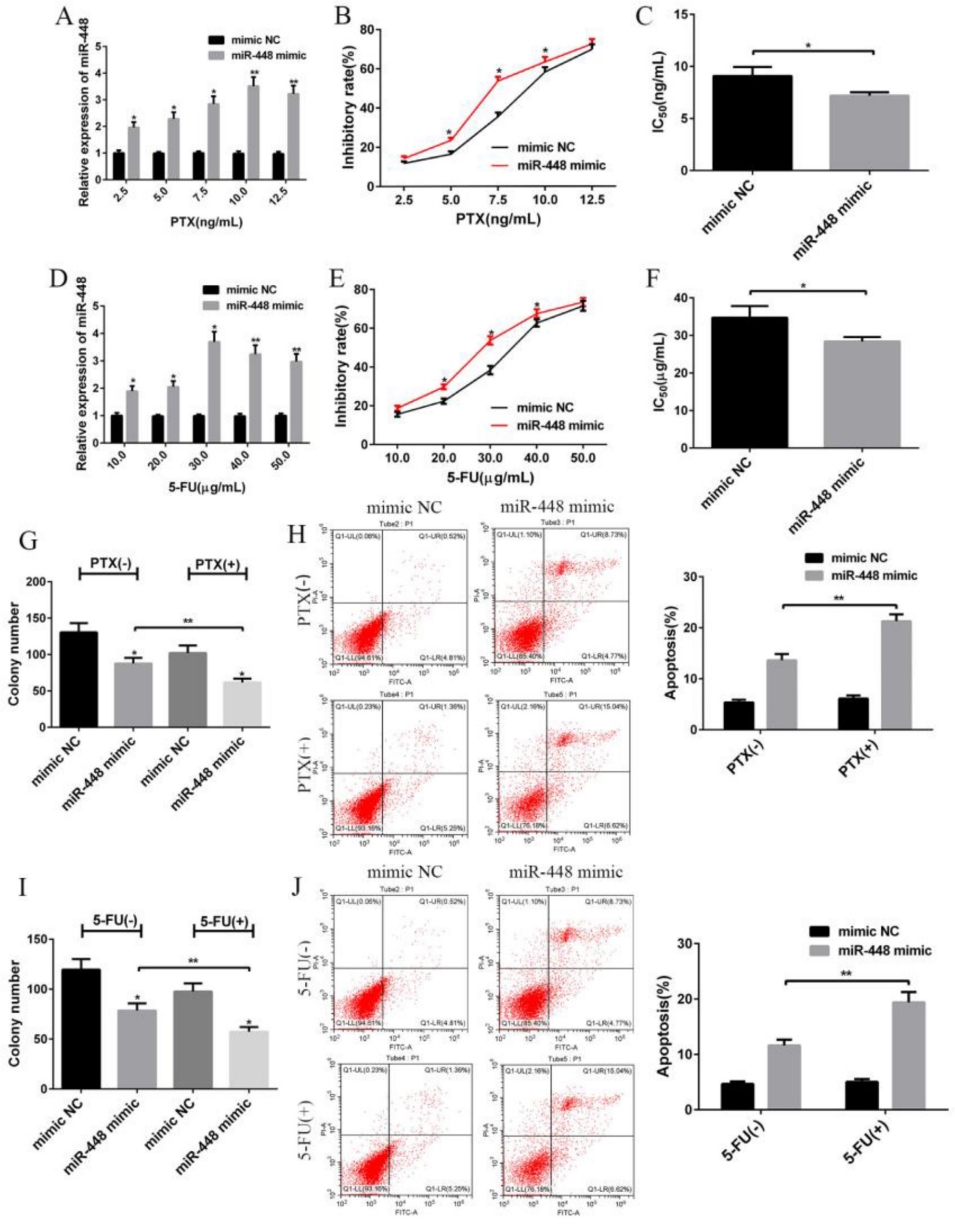
**Influence of miR-448 on the sensitivity of BC cells to paclitaxel and 5-fluorouracil.** (A) Comparison of transfection efficiency of miR-448 with different concentrations of paclitaxel; (B) Growth inhibition rates of cells following treatment with different concentrations of paclitaxel; (C) IC50 value of paclitaxel in each group of cells; (D) Comparison of transfection efficiency of miR-448 with different concentrations of 5-fluorouracil; (E) Growth inhibition rates of cells following treatment with different concentrations of 5-fluorouracil; (F) IC50 value of 5-fluorouracil in cells of each group; (G) miR-448 enhances paclitaxel-induced inhibition of MCF-7 cell proliferation; (H) miR-448 enhances the apoptosis-promoting effects of paclitaxel on MCF-7 cells; (I) miR-448 enhances 5-fluorouracil-induced inhibition of MCF-7 cell proliferation; (J) miR-448 enhances MCF-7 cell apoptosis promoted by 5-fluorouracil; * Compared with the control group, p < 0.05; ** p < 0.01.

Different concentrations of PTX and 5-FU had different effects on miR-448 transfection efficiency. The results showed that the optimal concentration of PTX was 10 ng/mL, while that of 5-FU was 30 μg/mL; these concentrations were used for subsequent experiments. The inhibition rate and IC50 values of PTX and 5-FU in each group were calculated. The results showed that transfection with the miR-448 mimic increased the inhibition rate of PTX and 5-FU in MCF-7 cells and decreased the IC50 value (p < 0.05, Fig. 5A‒5F). Thus, miR-448 can increase the sensitivity of MCF-7 cells to PTX and 5-FU. The plate cloning results revealed that miR-448 combined with PTX or 5-FU reduced the number of plate clones (p < 0.01, Fig. 5G, 5I), inhibited proliferation and increased the apoptosis rate (p < 0.01, Fig. 5H, 5J). This indicated that miR-448 could increase the inhibitory effects of PTX and 5-FU on MCF-7 cells and accelerate apoptosis (Fig. 5).

### Influence of miR-448 and ZEB1 on the sensitivity of BC cells to PTX and 5-FU

**Fig. 6:**
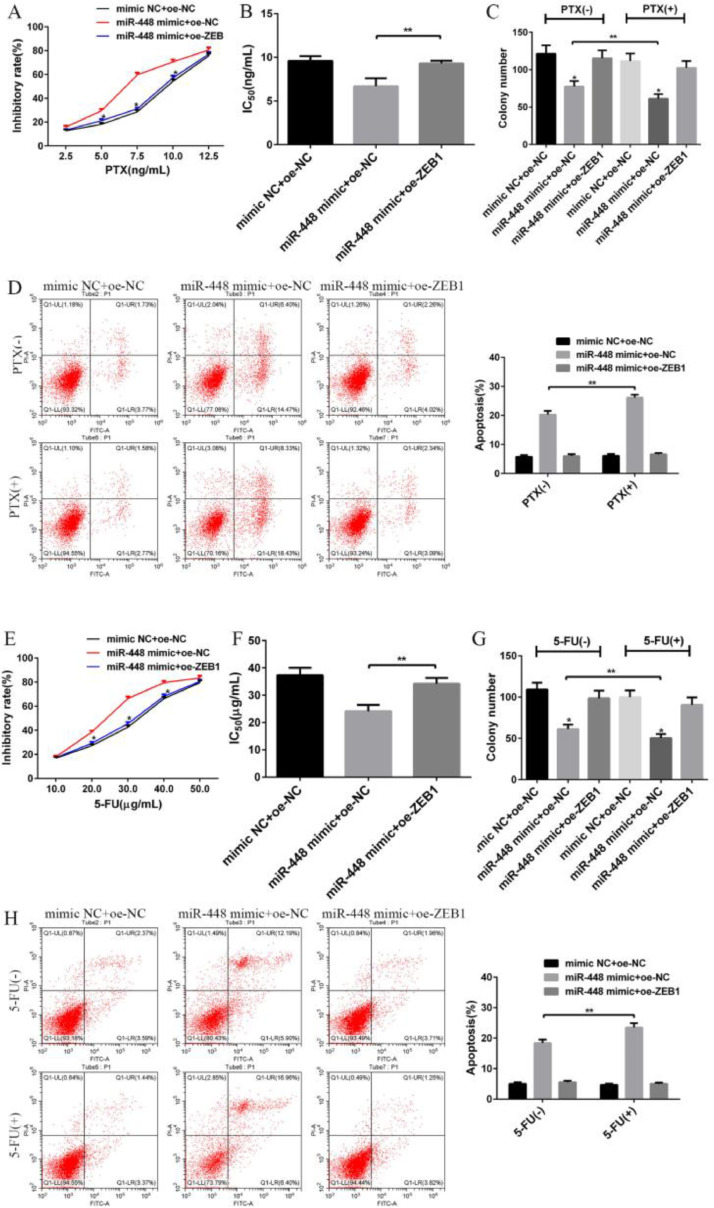
**Influence of miR-448 and ZEB1 on the sensitivity of BC cells to paclitaxel and 5-fluorouracil.** (A) Growth inhibition rates of cells from each group following treatment with different concentrations of paclitaxel; (B) IC50 value of paclitaxel in cells of each group; (C) Effects of miR-448 and ZEB1 combined with paclitaxel on the proliferation of MCF-7 cells; (D) Effects of miR-448 and ZEB1 combined with paclitaxel on the apoptosis of MCF-7 cells; (E) Growth inhibition rates of cells from each group following treatment with different concentrations of 5-fluorouracil; (F) IC50 value of 5-fluorouracil in cells of each group; (G) Effects of miR-448 and ZEB1 combined with 5-fluorouracil on the proliferation of MCF-7 cells; (H) Effects of miR-448 and ZEB1 combined with 5-fluorouracil on MCF-7 cell apoptosis; * Compared with the control group, p < 0.05; **p < 0.01.

Compared with the miR-448 mimic+oe-NC group, miR-448 mimic+oe-ZEB1 reduced the inhibition rate of PTX and 5-FU in MCF-7 cells and increased its IC50 value (p < 0.05, Fig. 5A‒5B,5E‒5F). Thus, miR-448 mimic+oe-ZEB1 can reduce the sensitivity of MCF-7 cells to PTX and 5-FU. The plate cloning results showed that miR-448 mimic+oe-ZEB1 combined with PTX or 5-FU increased the number of plate clones (p < 0.01, Fig. 5C, 5G), promoted cell proliferation, and decreased the apoptosis rate (p < 0.01, Fig. 5D, 5H). Therefore, miR-448 mimic+oe-ZEB1 can reduce the inhibitory effects of PTX and 5-FU on MCF-7 cells and delay apoptosis (Fig. 6).

## Discussion

Imbalance in miRNA expression is associated with the development and progression of almost all types of human cancers, affecting cancer cell proliferation, apoptosis, metastasis, angiogenesis, and chemosensitivity as well as the characteristics of cancer stem cells.^19^ Recently, miRNAs have attracted attention as participants in tumor chemotherapy sensitivity. Pan et al.^20^ confirmed that miR-503 enhanced MCF-7/ADR cell chemosensitivity by inhibiting EIF4G expression. Chen et al.^21^ discovered that miR-504 is involved in cisplatin resistance in MG63 osteosarcoma cells by inhibiting p53. In BC treatment, several miRNAs, such as miR-216b,^22^ miR-93,^23^ and miR-129-5p,^24^ have been found to be involved in the regulation of chemotherapy sensitivity.

Anthracyclines and taxanes have been widely used for BC adjuvant therapy.^25^ PTX is an antitumor drug that has been used as a chemotherapeutic drug against many cancers because of its microtubule-stabilizing effects.^26^ Many studies have also shown its use as an antitumor drug.^27^,^28^ In addition, it has different mechanisms of action at different stages of tumor development. For example, it can activate a cascade of signaling pathways, leading to apoptosis.^29^,^30^ PTX can also influence the development of tumors by regulating miRNA expression. One of the most commonly used cytotoxic drugs, 5-FU, can be used for treating various malignancies, especially advanced gastrointestinal cancer and BC.^31^,^32^ Metabolites of 5-FU destroy the synthesis of DNA and RNA through thymidylate synthase.^33^,^34^ The combination of 5-FU and other chemotherapeutic drugs is often used in the standard chemotherapy regimen for various solid tumors (including BC).^35^ Studies have shown that 5-FU combined with other drugs can also induce apoptosis of human gummed paper cells through autophagy. However, due to the imbalance of signaling pathway expression, mutation of genes, or epigenetic influence, BC cells are resistant to PTX and 5-FU, which leads to poor clinical outcomes.

The results showed that miR-448 expression in BC tissues was lower than that in normal tissues, while ZEB1 expression was increased. In addition, ZEB1 was lower in BC tissues with lymph node metastasis than in those without metastasis. In patients with clinical stage I–III BC, miR-448 expression decreased with an increase in tumor stage, which was negatively correlated with ZEB1 expression. Some studies have found that miR-448 expression is downregulated in many cancers, such as colon ^36^ and lung cancers.^33^ miR-448 expression is also downregulated during EMT induced by BC chemotherapy.^34^ A previous study reported that in BC, ZEB1 expression could reduce the expression of inflammatory cytokines,^35^ which is similar to the findings of the present study. Upregulation of miR-448 expression can suppress MCF-7 cell proliferation and invasion and promote apoptosis. Upregulation of ZEB1 expression in cells overexpressing miR-448 can partially reverse miR-448-induced inhibition of BC cell growth. Peng et al.^37^ found that ectopic miR-448 expression suppresses cancer cell migration and invasion by regulating the EMT. Their results were verified in the present study. miRNAs regulate the sensitivity of cancer cells toward PTX and 5-FU. Mao et al.^38^ and Li et al.^39^ confirmed that PTX could increase the ratio of CD44+/CD24- cells in BC. Collectively, the findings of these studies suggest that the enrichment of cancer stem cells induced by PTX may be a vital reason for the decrease in drug sensitivity. It has been reported that miRNAs, such as miR-1204,^40^ miR-125b,^41^ and miR-634,^42^ can increase sensitivity to PTX. It has also been reported that miRNAs, such as miR-489 ^43^ and miR-210,^44^ can increase the drug sensitivity of 5-FU. The current study demonstrated that miR-448 could enhance the sensitivity of BC cells toward PTX and 5-FU.

However, there are a few limitations to the study. First, there may exist other molecules in micro ribonucleic acid-448 that regulates zinc finger e-box binding homeobox 1 to inhibit the growth of breast cancer cells and increase their sensitivity to chemotherapy which may have the same effects. Second, breast cancer cells animal models must be created to verify the present research findings in vitro. Third, more clinical data needs be collected to confirm the importance of micro ribonucleic acid-448.

In summary, miR-448 expression decreased in BC tissues. Upregulation of miR-448 expression inhibits cancer cell proliferation and invasion, promotes apoptosis by targeting ZEB1, and increases the sensitivity of the cells to PTX or 5-FU. This indicates that upregulation of miR-448 expression is an effective method to enhance the efficacy of BC treatment.

## Authors' contributions

Yizhou Zhou, Li Sun, Yangmei Zhang and Kai Chen conceived the project, designed, and performed the experiments, analyzed the data. Yizhou Zhou and Kai Chen wrote and revised the manuscript.

## Funding

This research did not receive any specific grant from funding agencies in the public, commercial, or not-for-profit sectors.

## Declaration of Competing Interest

The authors declare no conflicts of interest.

## References

[1] Smith R.A., Manassaram-Baptiste D., Brooks D., Doroshenk M., Fedewa S., Saslow D. (2015). Cancer screening in the United States, 2015: a review of current American cancer society guidelines and current issues in cancer screening. CA Cancer J Clin.

[2] Pan R., Zhu M., Yu C., Lv J., Guo Y., Bian Z. (2017). Cancer incidence and mortality: A cohort study in China, 2008-2013. Int J Cancer.

[3] Falkenberry S.S., Legare RD. (2002). Risk factors for breast cancer. Obstet Gynecol Clin North Am.

[4] Ghoncheh M., Momenimovahed Z., Salehiniya H. (2016). Epidemiology, incidence and mortality of breast cancer in Asia. Asian Pac J Cancer Prev.

[5] Al-Hilli Z., Boughey JC. (2016). The timing of breast and axillary surgery after neoadjuvant chemotherapy for breast cancer. Chin Clin Oncol.

[6] Gomes da Cunha J.P., Goncalves R., Silva F., Aguiar F.N., Mota B.S., Chequim B.B. (2021 Oct 7). Validation of the Residual Cancer Burden Index as a prognostic tool in women with locally advanced breast cancer treated with neoadjuvant chemotherapy. J Clin Pathol.

[7] Drăgănescu M., Carmocan C. (2017). Hormone therapy in breast cancer. Chirurgia (Bucur).

[8] Castaneda S.A., Strasser J. (2017). Updates in the treatment of breast cancer with radiotherapy. Surg Oncol Clin N Am.

[9] Gu G., Dustin D., Fuqua S.A. (2016). Targeted therapy for breast cancer and molecular mechanisms of resistance to treatment. Curr Opin Pharmacol.

[10] Si W., Shen J., Du C., Chen D., Gu X., Li C. (2018). A miR-20a/MAPK1/c-Myc regulatory feedback loop regulates breast carcinogenesis and chemoresistance. Cell Death Differ.

[11] Ganz P.A., Goodwin PJ. (2015). Breast cancer survivorship: where are we today?. Adv Exp Med Biol.

[12] Sopik V., Narod SA. (2018). The relationship between tumour size, nodal status and distant metastases: on the origins of breast cancer. Breast Cancer Res Treat.

[13] Arpino G., Pensabene M., Condello C., Ruocco R., Cerillo I., Lauria R. (2016). Tumor characteristics and prognosis in familial breast cancer. BMC Cancer.

[14] Qadir M.I., Faheem A. (2017). miRNA: a diagnostic and therapeutic tool for pancreatic cancer. Crit Rev Eukaryot Gene Expr.

[15] Mishra S., Yadav T., Rani V. (2016). Exploring miRNA based approaches in cancer diagnostics and therapeutics. Crit Rev Oncol Hematol.

[16] Sandiford O.A., Moore C.A., Du J., Boulad M., Gergues M., Eltouky H. (2018). Human aging and cancer: role of miRNA in tumor microenvironment. Adv Exp Med Biol.

[17] Liang Z., Wu H., Xia J., Li Y., Zhang Y., Huang K. (2010). Involvement of miR-326 in chemotherapy resistance of breast cancer through modulating expression of multidrug resistance-associated protein 1. Biochem Pharmacol.

[18] Gu X., Xue J.Q., Han S.J., Qian S.Y., Zhang WH. (2016). Circulating microRNA-451 as a predictor of resistance to neoadjuvant chemotherapy in breast cancer. Cancer Biomark.

[19] Takahashi R.U., Prieto-Vila M., Kohama I., Ochiya T. (2019). Development of miRNA-based therapeutic approaches for cancer patients. Cancer Sci.

[20] Pan X., Yang X., Zang J., Zhang S., Huang N., Guan X. (2017). Downregulation of eIF4G by microRNA-503 enhances drug sensitivity of MCF-7/ADR cells through suppressing the expression of ABC transport proteins. Oncol Lett.

[21] Chen X., Lv C., Zhu X., Lin W., Wang L., Huang Z. (2019). MicroRNA-504 modulates osteosarcoma cell chemoresistance to cisplatin by targeting p53. Oncol Lett.

[22] Zou J., Kuang W., Hu J., Rao H. (2017). miR-216b promotes cell growth and enhances chemosensitivity of colorectal cancer by suppressing PDZ-binding kinase. Biochem Biophys Res Commun.

[23] Bao C., Chen J., Chen D., Lu Y., Lou W., Ding B. (2020). MiR-93 suppresses tumorigenesis and enhances chemosensitivity of breast cancer via dual targeting E2F1 and CCND1. Cell Death Dis.

[24] Yi H., Liu L., Sheng N. (2016). Synergistic therapy of doxorubicin and miR-129-5p with self-cross-linked bioreducible polypeptide nanoparticles reverses multidrug resistance in cancer cells. Biomacromolecules.

[25] Zaheed M., Wilcken N., Willson M.L., O'Connell D.L., Goodwin A. (2019). Sequencing of anthracyclines and taxanes in neoadjuvant and adjuvant therapy for early breast cancer. Cochrane Database Syst Rev.

[26] Weaver BA. (2014). How Taxol/paclitaxel kills cancer cells. Mol Biol Cell.

[27] Ezrahi S., Aserin A., Garti N. (2019). Basic principles of drug delivery systems ‒ the case of paclitaxel. Adv Colloid Interface Sci.

[28] Mitchison T.J., Pineda J., Shi J., Florian S. (2017). Is inflammatory micronucleation the key to a successful anti-mitotic cancer drug?. Open Biol.

[29] Abu Samaan T.M., Samec M., Liskova A., Kubatka P., Büsselberg D. (2019). Paclitaxel's mechanistic and clinical effects on breast cancer. Biomolecules.

[30] Kawiak A., Domachowska A., Lojkowska E. (2019). Plumbagin increases paclitaxel-induced cell death and overcomes paclitaxel resistance in breast cancer cells through ERK-mediated apoptosis induction. J Nat Prod.

[31] Lu Y., Zhang R., Zhang X., Zhang B., Yao Q. (2020). Curcumin may reverse 5-fluorouracil resistance on colonic cancer cells by regulating TET1-NKD-Wnt signal pathway to inhibit the EMT progress. Biomed Pharmacother.

[32] Wińska P., Karatsai O., Staniszewska M., Koronkiewicz M., Chojnacki K., Rędowicz MJ. (2020). Synergistic interactions of 5-fluorouracil with inhibitors of protein kinase CK2 correlate with p38 MAPK activation and FAK inhibition in the triple-negative breast cancer cell line. Int J Mol Sci.

[33] Liu H.Y., Chang J., Li G.D., Zhang Z.H., Tian J., Mu YS. (2020). MicroRNA-448/EPHA7 axis regulates cell proliferation, invasion and migration via regulation of PI3K/AKT signaling pathway and epithelial-to-mesenchymal transition in non-small cell lung cancer. Eur Rev Med Pharmacol Sci.

[34] Li Q.Q., Chen Z.Q., Cao X.X., Xu J.D., Xu J.W., Chen Y.Y. (2011). Involvement of NF-κB/miR-448 regulatory feedback loop in chemotherapy-induced epithelial-mesenchymal transition of breast cancer cells. Cell Death Differ.

[35] Katsura A., Tamura Y., Hokari S., Harada M., Morikawa M., Sakurai T. (2017). ZEB1-regulated inflammatory phenotype in breast cancer cells. Mol Oncol.

[36] Lou Q., Liu R., Yang X., Li W., Huang L., Wei L. (2019). MiR-448 targets IDO1 and regulates CD8(+) T cell response in human colon cancer. J Immunother Cancer.

[37] Ma P., Ni K., Ke J., Zhang W., Feng Y., Mao Q. (2018). MiR-448 inhibits the epithelial-mesenchymal transition in breast cancer cells by directly targeting the E-cadherin repressor ZEB1/2. Exp Biol Med (Maywood).

[38] Mao J., Song B., Shi Y., Wang B., Fan S., Yu X. (2013). ShRNA targeting Notch1 sensitizes breast cancer stem cell to paclitaxel. Int J Biochem Cell Biol.

[39] Li H.Z., Yi T.B., Wu ZY. (2008). Suspension culture combined with chemotherapeutic agents for sorting of breast cancer stem cells. BMC Cancer.

[40] Peng X., Cao P., Li J., He D., Han S., Zhou J. (2015). MiR-1204 sensitizes nasopharyngeal carcinoma cells to paclitaxel both in vitro and in vivo. Cancer Biol Ther.

[41] Zhang Y., Huang S. (2015). Up-regulation of miR-125b reverses epithelial-mesenchymal transition in paclitaxel-resistant lung cancer cells. Biol Chem.

[42] van Jaarsveld M.T., van Kuijk P.F., Boersma A.W., Helleman J., van IJcken W.F., Mathijssen R.H.J. (2015). miR-634 restores drug sensitivity in resistant ovarian cancer cells by targeting the Ras-MAPK pathway. Mol Cancer.

[43] Wang X., Wang X., Gu J., Zhou M., He Z., Wang X. (2017). Overexpression of miR-489 enhances efficacy of 5-fluorouracil-based treatment in breast cancer stem cells by targeting XIAP. Oncotarget.

[44] Yang Y.F., Xiao Y.Z., Duan S.Y., Luan ZH. (2018). MiR-210 promotes 5-fluorouracil resistance in breast cancer cells by increasing the antioxidant activity mediated by GPX1. Zhonghua Bing Li Xue Za Zhi.

